# Preoperative asymptomatic leukocytosis and postoperative outcome in cardiac surgery patients

**DOI:** 10.1371/journal.pone.0182118

**Published:** 2017-09-05

**Authors:** Eitezaz Mahmood, Ziyad O. Knio, Feroze Mahmood, Rabia Amir, Sajid Shahul, Bilal Mahmood, Yanick Baribeau, Ariel Mueller, Robina Matyal

**Affiliations:** 1 Department of Anesthesia, Critical Care and Pain Medicine, Beth Israel Deaconess Medical Center, Harvard Medical School, Boston, Massachusetts, United States of America; 2 Feinberg School of Medicine, Northwestern University, Chicago, Illinois, United States of America; 3 Department of Surgery, Division of Cardiac Surgery, Beth Israel Deaconess Medical Center, Harvard Medical School, Boston, Massachusetts, United States of America; 4 Albany Medical College, Albany, New York, United States of America; University of Milano, ITALY

## Abstract

**Background:**

Despite showing a prognostic value in general surgical patients, preoperative asymptomatic elevated white blood cell (WBC) count is not considered a risk factor for cardiac surgery. Whereas there is sporadic evidence of its value as a preoperative risk marker, it has not been looked at methodically as a specific index of outcome during cardiac surgery. Using a national database we sought to determine the relationship between preoperative WBC count and postoperative outcome in cardiac surgical patients.

**Methods:**

Cardiac surgeries were extracted from the 2007–2013 American College of Surgeons National Surgical Quality Improvement Program database. Leukocytosis was defined by a preoperative WBC count greater than 11,000 cells/μL. A univariate analysis compared the incidence of adverse outcomes for patients with and without leukocytosis. A multivariate logistic regression model was constructed in order to test whether leukocytosis was an independent predictor of morbidity and mortality.

**Results:**

Out of a total of 10,979 cardiac surgery patients 863 (7.8%) had preoperative leukocytosis. On univariate analysis, patients with leukocytosis experienced greater incidences of 30-day mortality, wound complications, and medical complications. Wound complications included surgical site infection as well as wound dehiscence. The medical complications included all other non-surgical causes of increased morbidity and infection leading to urinary tract infection, pneumonia, ventilator dependence, sepsis and septic shock. After stepwise model adjustment, leukocytosis was a strong predictor of medical complications (OR 1.22, 95% CI: 1.09–1.36, p = 0.002) with c-statistic of 0.667. However, after stepwise model adjustment leukocytosis was not a significant predictor of 30-day mortality and wound complications.

**Conclusion:**

Preoperative leukocytosis is associated with adverse postoperative outcome after cardiac surgery and is an independent predictor of infection-related postoperative complications.

## Introduction

Leukocytosis is a non-specific marker for a systemic inflammatory state [1,2]. While systemic inflammation is a biological protective response, it is associated with humoral and cellular components that result in vascular injury and eventual organ dysfunction [3,4]. Surgical trauma induced non-specific inflammation is possibly the “second hit” in patients with pre-existing systemic inflammatory state plausibly associated with adverse outcome [2]. Systemic inflammation after cardiac surgery is a well-known phenomenon that is attributable to various mechanisms [5]. This vigorous non-specific inflammatory state with clinical sequel is often referred to as the systemic inflammatory response syndrome (SIRS) [6–9]. Leukocytosis, defined as a white blood cell (WBC) count greater than 11,000 cells per microliter, is amongst the criteria used for diagnosing SIRS [1]. Multiple etiological factors are believed to play a role in the initiation and perpetuation of SIRS, such as patient exposure to cardiopulmonary bypass, and ischemia-reperfusion injury which may lead to vascular injury via neutrophil-endothelial interactions [2,10,11].

Despite the implementation of “fast track” pathways and rigorous antimicrobial coverage for cardiac surgical patients, the incidence of postoperative pneumonia and systemic infection has not decreased [12]. While multiple focused studies have demonstrated the value of leukocytosis as a marker of specific outcomes such as morbidity and mortality following percutaneous coronary interventions and CABG for acute coronary syndromes [13–24], these studies did not exclude known causes of leukocytosis which limited their ability to conclude a direct relationship between leukocytosis and adverse postoperative outcomes. As such, an elevated WBC count is not considered a risk factor in the adult Society of Thoracic Surgeons (STS) database.

In this study we queried the American College of Surgeons National Quality Improvement Program (ACS NSQIP) database to assess the association between preoperative leukocytosis and postoperative morbidity and mortality.

## Materials and methods

### Cohort assembly

The ACS NSQIP database was used to investigate the predictive ability of leukocytosis on 30-day mortality, wound complications, and medical complications following cardiac surgery.

The ACS NSQIP includes data on over 300 total variables describing the patient demographic and preoperative/intraoperative/postoperative data associated with a case. Definitions and protocols are explained in the ACS NSQIP Participant Use File. For the purpose of this study, leukocytosis was characterized by a preoperative WBC count greater than 11,000 cells per microliter [10,25,26]. Wound complications were defined as postoperative complications that involved the incision site, and included superficial surgical site infections, deep wound infections, organ/space infections, and wound dehiscence. All remaining postoperative complications were considered to be medical complications and included instances of unplanned reintubation, pulmonary embolism, ventilator dependence, renal insufficiency, renal failure, urinary tract infection, stroke, coma, cardiac arrest, myocardial infarction, pneumonia, deep vein thrombosis, sepsis, and septic shock. Additional variable definitions, compositions, and modifications pertinent to this specific study are detailed in S1 Table.

As all patient data was de-identified prior to author access, and contained no protected health information, the Institutional Review Board at Northwestern University assigned a determination of Not Human Research and approved a waiver of informed consent for this study. Using the 2007–2013 ACS NSQIP database, cardiac surgeries were extracted by querying for cases in which the primary surgeon’s specialty was “cardiac surgeon.” Patients presenting with preoperative sepsis, septic shock, systemic inflammatory response syndrome, pneumonia, wound infections, disseminated cancer, renal failure, or a history of chronic steroid use were excluded from the study.

### Statistical methods

Differences in demographic, intraoperative, and postoperative data were compared between patients with and without leukocytosis. Unadjusted data are presented as mean ± standard deviation or frequency and proportion depending on variable type (quantitative or categorical) and assessed using t-tests or chi-square tests, respectively.

Postoperative outcomes/complications of interest in this study included 30-day mortality, a composite outcome for wound complications, and a composite outcome for medical complications. Multivariable logistic regression models were constructed for the three outcomes to assess for independent risk factors for each of these outcomes.

Potential risk factors investigated included: age, preoperative creatinine, sex, American Society of Anesthesiologists (ASA) class, obesity, diabetes, hypertension, smoking status, alcohol consumption, dyspnea, angina, emergency procedure, history of chronic obstructive pulmonary disease (COPD), history of congestive heart failure (CHF), history of myocardial infarction (MI), history of peripheral vascular disease (PVD), and previous cardiac procedure. ASA class was intended to be considered a potential risk factor for the subsequent multiple logistic regression models. However, it was neglected because of the apparent over-representation of patients with an ASA grade of 3 or greater (98.4%) in this subset of cardiac surgery patients.

All risk factors with p-values less than 0.10 on univariate analyses were considered as covariates and entered in a bidirectional stepwise logistic regression model selection algorithm using the Akaike Information Criteria (AIC) cutoff for inclusion. The R software (Version 3.3.0., R Core Team, Vienna, Austria) accomplished this by algorithmically adding and removing covariates until the model fit (as measured by AIC) and could no longer be improved with additional iterations. Cases with missing data were excluded from the model selection procedure entirely.

The accuracy of each of these models was assessed by its calibration and discrimination, measured by the Hosmer-Lemeshow hypothesis test and a c-statistic, respectively.

## Results

A total of 10,979 cardiac surgery patients were included in our analysis, of whom 863 (7.8%) had asymptomatic leukocytosis. Of the 10,979 cases, 5384 (48.7%) were CABG procedures, 3136 (28.6%) were valvular procedures, 1093 (10.0%) were combined CABG/valvular procedures, and 1402 (12.8%) were other cardiac procedures.

### Comparisons between patients with and without leukocytosis

Patients with leukocytosis were younger, presenting with higher creatinine, and more often obese, diabetic, smokers, presenting with dyspnea and angina, undergoing an emergent procedure, and presenting with history of COPD, CHF, and MI. There was no significant difference in gender, alcohol consumption, incidence of hypertension, history of PVD, or incidence of previous cardiac procedure between the two groups (Table 1).

**Table 1:** Univariate associations between leukocytosis and baseline characteristics.

Variable	WBC<11k		WBC> = 11k		n	p-value	
	n		n				
	10116		863		10979		
Age	65.644	12.621	63.563	12.758	10979	<0.001	*
Creatinine	1.115	0.808	1.184	0.932	10931	0.038	*
Sex (male)	6855/10096	67.9%	579/858	67.5%	10954	0.832	
ASA Grade 3 or Greater	9937/10102	98.4%	853/860	99.2%	10962	0.087	
Obesity (BMI>30)	3831/9968	38.4%	354/841	42.1%	10809	0.040	*
Diabetes	2698/10116	26.7%	263/863	30.5%	10979	0.017	*
Hypertension	7735/10116	76.5%	647/863	75.0%	10979	0.343	
Tobacco smoker	1717/10116	17.0%	306/863	35.5%	10979	<0.001	*
Alcohol consumption	454/10116	4.5%	42/863	4.9%	10979	0.668	
Dyspnea	5397/10116	53.4%	400/863	46.3%	10979	<0.001	*
Angina	3546/10116	35.1%	365/863	42.3%	10979	<0.001	*
Emergency Procedure	596/10116	5.9%	203/863	23.5%	10979	<0.001	*
History of COPD	836/10116	8.3%	116/863	13.4%	10979	<0.001	*
History of CHF	1288/10116	12.7%	151/863	17.5%	10979	<0.001	*
History of MI	1659/10116	16.4%	267/863	30.9%	10979	<0.001	*
History of PVD	335/10116	3.3%	35/863	4.1%	10979	0.287	
Previous Cardiac Procedure	3091/10116	30.6%	252/863	29.2%	10979	0.428	

Univariate associations between leukocytosis (>11,000 white blood cell count per microliter) and baseline characteristics. Continuous variables are expressed as a mean and standard deviation, while categorical variables are expressed as a proportion and a percentage. BMI = Body Mass Index; COPD = Chronic Obstructive Pulmonary Disease; CHF = Congestive Heart Failure; MI = Myocardial Infarction; PVD = Peripheral Vascular Disease.

* indicates statistically significant p-values at the 0.05 significance level.

Patients with leukocytosis were in the hospital for a greater number of days (11.65 ± 8.72) as compared to patients without leukocytosis (9.88 ± 9.79; p<0.001). There was not a significant difference in operative time or incidence of blood transfusion between the two groups (Table 2).

**Table 2:** Univariate associations between leukocytosis and outcomes.

Variable	WBC<11k		WBC> = 11k		n	p-value	
	n		n				
	10116		863		10979		
Operative Time	263.150	103.621	259.424	111.314	10976	0.343	
Blood Transfusions	3311/10116	32.7%	303/863	35.1%	10979	0.164	
Total Hospital Stay (days)	9.878	9.786	11.648	8.722	10960	<0.001	
**30-Day Mortality**	**253/10116**	**2.5%**	**33/863**	**3.8%**	**10979**	**0.026**	*
**Wound Complications (composite)**	**405/10116**	**4.0%**	**47/863**	**5.4%**	**10979**	**0.050**	
Superficial Incisional SSI	311/10116	3.1%	31/863	3.6%	10979	0.460	
Deep Incisional SSI	44/10116	0.4%	5/863	0.6%	10979	0.730	
Organ/Space SSI	29/10116	0.3%	6/863	0.7%	10979	0.084	
Wound Disruption	42/10116	0.4%	11/863	1.3%	10979	0.001	*
**Medical Complications (composite)**	**1513/10116**	**15.0%**	**197/863**	**22.8%**	**10979**	**<0.001**	*
Unplanned Reintubation	343/10116	3.4%	39/863	4.5%	10979	0.101	
Pulmonary Embolism	60/10116	0.6%	10/863	1.2%	10979	0.075	
On Ventilator > 48 Hours	604/10116	6.0%	102/863	11.8%	10979	<0.001	*
Progressive Renal Insufficiency	90/10116	0.9%	11/863	1.3%	10979	0.341	
Acute Renal Failure	178/10116	1.8%	26/863	3.0%	10979	0.013	*
Urinary Tract Infection	197/10116	1.9%	19/863	2.2%	10979	0.698	
Stroke/CVA	171/10116	1.7%	24/863	2.8%	10979	0.028	*
Coma Greater Than 24 Hours	22/10116	0.2%	4/863	0.5%	10979	0.288	
Cardiac Arrest Requiring CPR	209/10116	2.1%	15/863	1.7%	10979	0.597	
Myocardial Infarction	54/10116	0.5%	6/863	0.7%	10979	0.706	
Pneumonia	365/10116	3.6%	54/863	6.3%	10979	<0.001	*
Deep Venous Thrombosis	149/10116	1.5%	25/863	2.9%	10979	0.002	*
Sepsis	170/10116	1.7%	26/863	3.0%	10979	0.007	*
Septic Shock	125/10116	1.2%	13/863	1.5%	10979	0.599	

Univariate associations between leukocytosis (>11,000 white blood cell count per microliter) and outcomes. Continuous variables are expressed as a mean and standard deviation, while categorical variables are expressed as a proportion and percentage. SSI = Surgical Site Infection; CVA = Cerebrovascular Accident; CPR: Cardiopulmonary Resuscitation

* indicates statistically significant p-values at the 0.05 significance level.

Patients with leukocytosis experienced greater incidences of 30-day mortality, wound disruption, and medical complications–specifically ventilator dependence, acute renal failure, stroke, pneumonia, deep vein thrombosis, and sepsis. No significant difference in the incidence of superficial infection, deep incisional infection, reintubation, renal insufficiency, urinary tract infection, coma, cardiac arrest, myocardial infarction, or septic shock was observed between groups (Table 2).

An association between 30-day mortality and leukocytosis was observed on the univariate level (p = 0.026). After adjustment for covariates, however, leukocytosis was no longer a significant independent predictor of 30-day mortality. The predictors of 30-day mortality were: age, history of CHF, previous cardiac procedure, emergency surgery, history of COPD, history of MI, preoperative creatinine, diabetes, and obesity.

Similarly, there was a marginal association between wound complications and leukocytosis on the univariate level (p = 0.050), however this did not persist after adjustment for covariates. The predictors of wound complications were: obesity, diabetes, angina, history of COPD, history of MI, preoperative creatinine, previous cardiac procedure, sex, history of PVD, and smoking status.

An association between medical complications and leukocytosis was observed on the univariate level (p<0.001). After adjustment for covariates, leukocytosis remained a significant independent predictor of medical complications (OR = 1.22, 95% CI 1.09–1.36; p = 0.002). The remaining predictors of medical complications were: history of CHF, age, emergency surgery, history of COPD, preoperative creatinine, sex, previous cardiac procedure, obesity, history of PVD, history of MI, and dyspnea.

**Fig 1:**
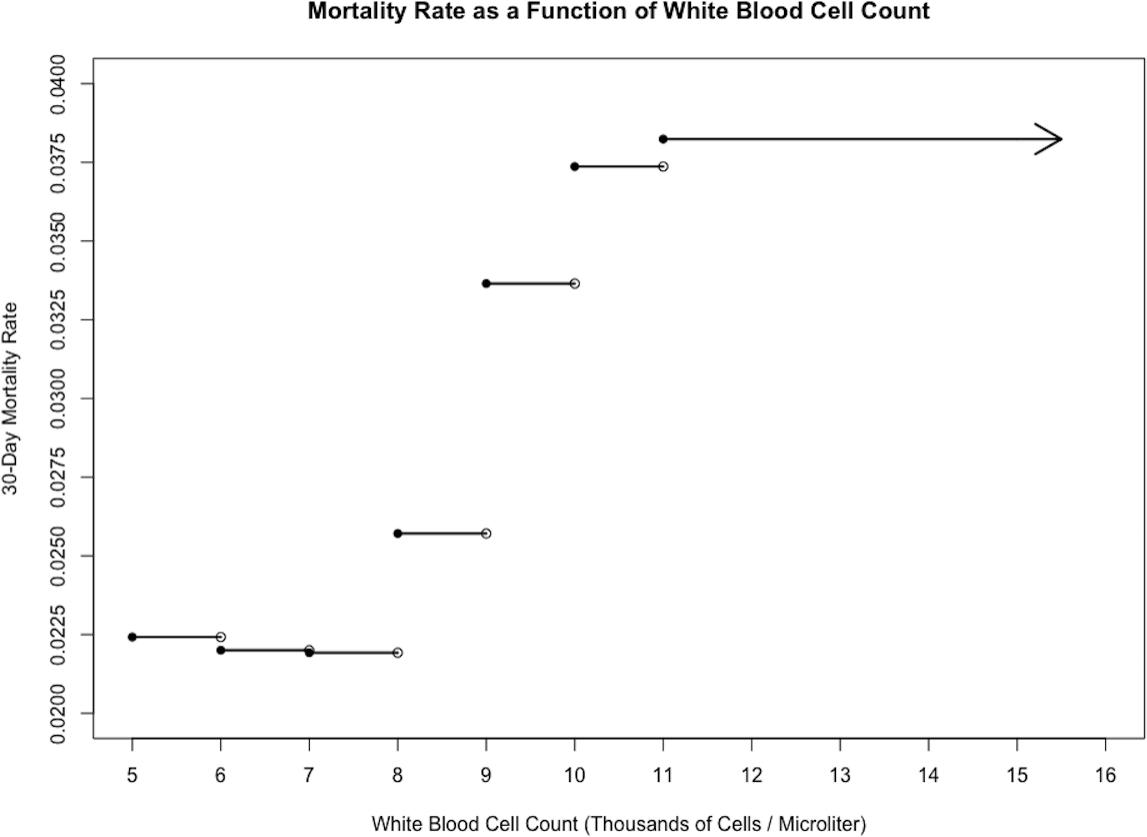
30-day mortality vs. white blood cell count. Stepwise plot of 30-day mortality rate against white blood cell count for the sample of 10,979 cardiac surgery patients, illustrating a positive relationship between the two variables.

**Fig 2:**
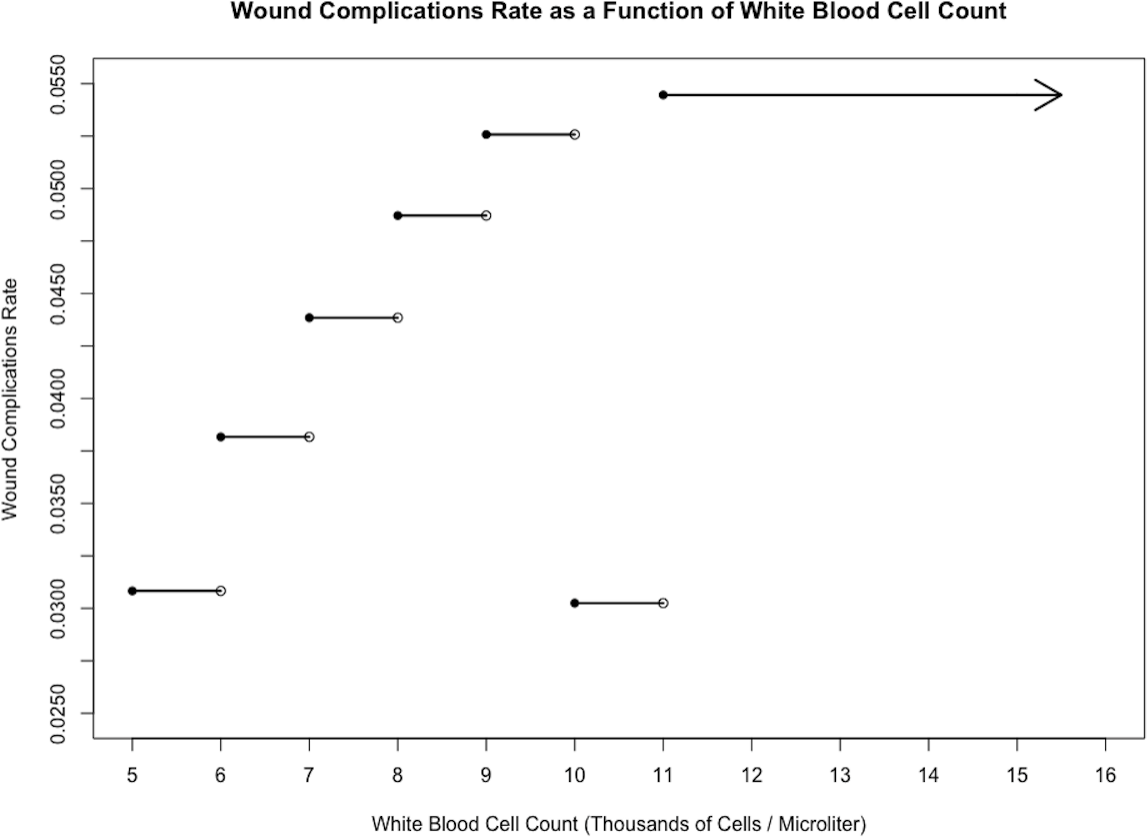
Wound complications vs. white blood cell count. Stepwise plot of wound complications rate against white blood cell count for the sample of 10,979 cardiac surgery patients, illustrating a positive relationship between the two variables.

**Fig 3:**
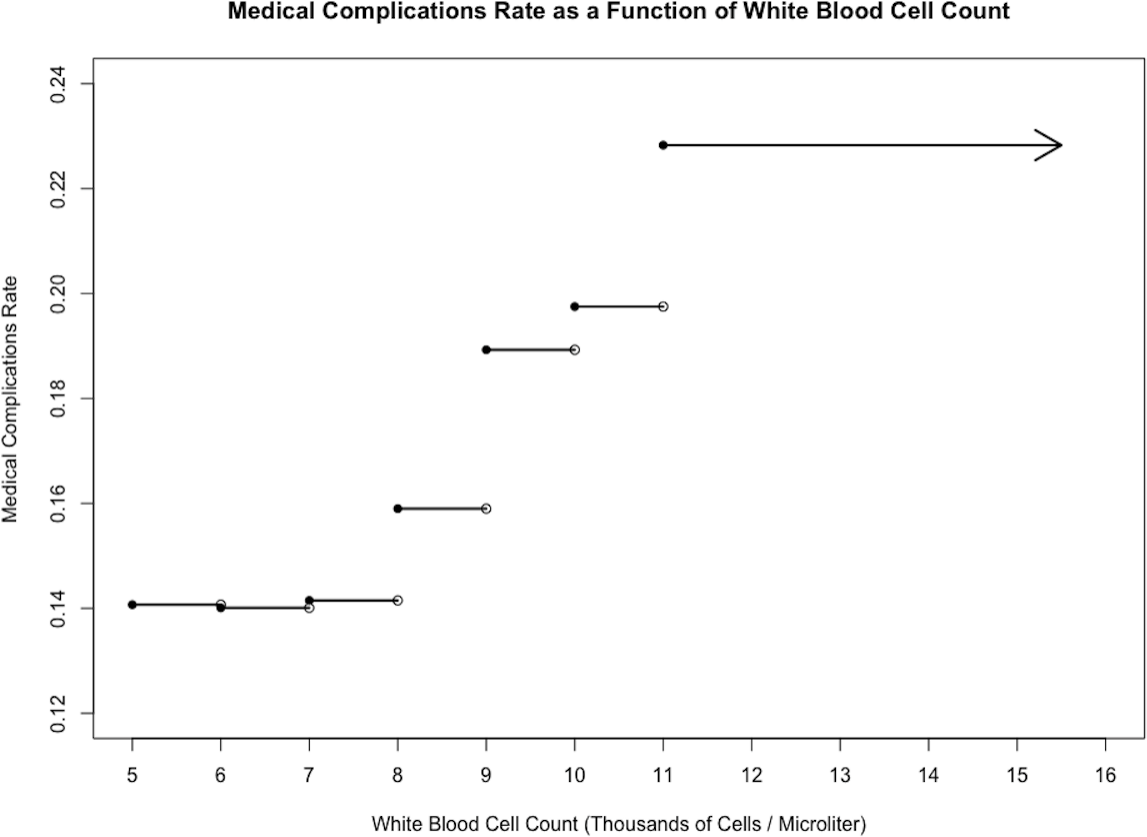
Medical complications vs. white blood cell count. Stepwise plot of medical complications rate against white blood cell count for the sample of 10,979 cardiac surgery patients, illustrating a positive relationship between the two variables.

Models for 30-day mortality, wound complications, and medical complications are reported in Table 3. Figs 1, 2 and 3 displays the relationship between WBC count and each outcome.

**Table 3:** Final multiple logistic regression model summaries.

Outcome	Predictor	p-value	Adjusted Odds Ratio	Lower Bound of 95% CI	Upper Bound of 95% CI	Hosmer-Lemeshow p	c-statistic
**30-Day Mortality**	(Intercept)	<0.001	0.001	0.000	0.001	0.040	0.745
	Age	<0.001	1.043	1.031	1.056		
	History of CHF	<0.001	1.959	1.473	2.582		
	Previous Cardiac Procedure	<0.001	1.801	1.408	2.302		
	Emergency Surgery	<0.001	2.339	1.626	3.291		
	History of COPD	<0.001	1.992	1.434	2.720		
	History of MI	<0.001	1.599	1.206	2.101		
	Preoperative Creatinine	<0.001	1.184	1.066	1.294		
	Diabetes	0.027	1.346	1.031	1.749		
	BMI>30	0.103	0.800	0.610	1.043		
**Wound Complications (composite)**	(Intercept)	<0.001	0.013	0.011	0.017	0.187	0.684
	BMI>30	<0.001	2.070	1.698	2.528		
	Diabetes	<0.001	1.701	1.391	2.077		
	Angina	<0.001	1.485	1.212	1.818		
	History of COPD	0.011	1.459	1.082	1.937		
	History of MI	0.010	1.355	1.073	1.702		
	Preoperative Creatinine	0.005	1.129	1.031	1.220		
	Previous Cardiac Procedure	0.025	1.257	1.028	1.534		
	Sex (female)	0.025	1.259	1.028	1.537		
	History of PVD	0.033	1.572	1.015	2.343		
	Smoker	0.090	1.227	0.965	1.549		
**Medical Complications (composite)**	(Intercept)	<0.001	0.017	0.012	0.023	0.122	0.667
	History of CHF	<0.001	1.845	1.603	2.122		
	Age	<0.001	1.024	1.020	1.029		
	Emergency Surgery	<0.001	2.025	1.684	2.426		
	History of COPD	<0.001	1.636	1.386	1.925		
	Preoperative Creatinine	<0.001	1.178	1.117	1.240		
	Sex (female)	<0.001	1.334	1.191	1.492		
	Previous Cardiac Procedure	<0.001	1.263	1.127	1.414		
	BMI>30	<0.001	1.377	1.144	1.651		
	Leukocytosis	<0.001	1.215	1.088	1.357		
	History of PVD	<0.001	1.548	1.196	1.986		
	History of MI	0.005	1.217	1.061	1.394		
	Dyspnea	0.067	1.112	0.993	1.246		

Final multiple logistic regression model summaries assessing for independent predictors for 30-day mortality, composite wound complications, and composite medical complications in 10,979 cardiac surgery patients. CHF = Congestive Heart Failure; COPD = Chronic Obstructive Pulmonary Disease; MI = Myocardial Infarction; BMI = Body Mass Index; PVD = Peripheral Vascular Disease.

## Discussion

Our study has demonstrated that asymptomatic leukocytosis is an independent predictor of morbidity but not mortality following cardiac surgery. The association between asymptomatic leukocytosis and the composite outcome of medical complications is of particular importance. These findings may point towards the possibility of an exaggerated subclinical inflammatory response. Preoperative leukocytosis may also be an independent marker of multi-organ dysfunction in cardiac surgery patients as evidenced by the significant association with acute renal failure and stroke in our study. Therefore, preoperative asymptomatic leukocytosis may warrant an additional level of scrutiny to improve morbidity in patients undergoing cardiac surgery.

Several recent studies have also shown an association between preoperative leukocytosis and postoperative infectious complications across a wide variety of other surgeries [27–29]. Postoperative pneumonia is the most common infection after cardiac surgery, and is a significant source of morbidity and mortality [12]. Strobel et.al recently developed a risk model for predicting pneumonia after cardiac surgery using the STS database, and concluded that preoperative leukocytosis may be an immune response to preexisting pathogens [30]. Previous studies have also found value in surveillance catheter cultures of intubated patients in anticipating ventilator-associated pneumonia [10]. While preoperative leukocytosis has been associated with an increased risk of ischemic stroke, acute cardiovascular events, atrial fibrillation, and new onset congestive heart failure in various studies [20,31–34], these studies are limited to specific patient populations and do not have the statistical power to provide a more nuanced assessment of leukocytosis and outcomes following cardiac surgery [16–19,21–24,35]. The largest study by Dacey et. al. examined 11,270 CABG surgery patients, finding leukocytosis to be an independent predictor of mortality and other adverse outcomes[20]. However, this study did not exclude patients who had other known causes of leukocytosis, such as sepsis, SIRS, wound infection, pneumonia, among others. This limited the ability for the study to draw a cause and effect relationship between leukocytosis and adverse outcomes. Despite the robustness of the correlation between leukocytosis and morbidity and mortality, strong arguments could be made that leukocytosis is a nonspecific marker of another underlying process that is increasing the risk of postoperative complications. Differently from previous studies, leukocytosis was found not to be an independent predictor of 30-day mortality, which was better predicted by the other risk factors that our study investigated (Table 3). Moreover, the exclusion criteria (patients with preoperative sepsis, septic shock, systemic inflammatory response syndrome, pneumonia, wound infections, disseminated cancer, renal failure, or a history of chronic steroid use) allowed our study to be unique. This was accomplished by excluding known causes of leukocytosis and the influence of confounding conditions with poor prognoses, in order to better demonstrate the true predictive ability of an asymptomatic elevated WBC count on adverse outcomes.

Incorporating WBC count as a risk marker can have significant clinical implications. While there is value in incorporating leukocytosis into surgical risk assessment models in order to improve risk assessment, the major advantage of this information may potentially lie in mitigating the inflammatory response. Non-pharmacologic techniques may include reducing surgical trauma and minimizing exposure to extracorporeal circulation systems. Similarly, hemofiltration and leukocyte depletion have been shown to suppress CPB induced SIRS [5]. Pharmacologic anti-inflammatory therapies may also help by targeting the coagulation and complement cascades, reducing oxidative stress, and decreasing cellular activation. Although, steroids have been shown to not have a significant effect on mortality or major morbidity after cardiac surgery with cardiopulmonary bypass [36], Dieleman et al have demonstrated a reduction of postoperative respiratory failure and duration of postoperative hospital stay following the use of intraoperative steroids in patients undergoing cardiac surgery [37]. Understanding the risk of a high WBC count before cardiac surgery may lead to additional therapies or surveillance strategies to mitigate risk of adverse outcomes.

Our study has several limitations. First, there is a lack of long-term follow-up with patients after 30 days and lack of differential WBC count. Moreover, the variables that were studied were limited strictly to those made available by the ACS NSQIP database. Therefore, it is possible that some causes of chronic inflammation that may predispose to leukocytosis such as chronic rheumatoid arthritis may have been included in our analysis, and some relevant risk factors such as ejection fraction could not be studied. The risk factors and outcomes in this study were selected based on the recommendations of prior studies and existing literature regarding WBC count and surgical outcomes; as such, the relevance of these risk factors and outcomes can be debated. Finally, although it is highly unlikely, some patients with undocumented infections may have been missed during our initial analysis. Finally, the number of patients with leukocytosis was not large enough to identify the trend in outcome with differential leukocytosis.

## Conclusion

In conclusion, our retrospective analysis of a large national database suggests that an elevated preparative WBC count is a strong independent predictor of adverse postoperative outcomes, especially pneumonia and prolonged ventilation, after cardiac surgery. Based on this observation it is reasonable to recommend utilization of a routine preoperative hematological evaluation as a risk assessment tool for cardiac surgery.

## Supporting information

S1 Table Additional variable information. Here, variables are classified, their database name as it appears in the 2007–2013 ACS NSQIP is indicated, and any conditions or modifications are explained. ASA = American Society of Anesthesiologists; COPD = Chronic Obstructive Pulmonary Disease; PVD = Peripheral Vascular Disease.
(DOCX)
