# Supplementary material for: Preoperative asymptomatic leukocytosis and postoperative outcome in cardiac surgery patients
Source: PLoS One. 2017 Sep 5;12(9):e0182118. doi: 10.1371/journal.pone.0182118 (PMC5584953; doi:10.1371/journal.pone.0182118)
Supplement: S1 Table — Here, variables are classified, their database name as it appears in the 2007–2013 ACS NSQIP is indicated, and any conditions or modifications are explained. ASA = American Society of Anesthesiologists; COPD = Chronic Obstructive Pulmonary Disease; PVD = Peripheral Vascular Disease. (DOCX) [file pone.0182118.s001.docx]

| **Class** | **Variable** | | **Database Name** | **Conditions Kept / Modifications** |
| --- | --- | --- | --- | --- |
| **Exclusion** | Leukocytosis | | PRWBC | "-99" is removed |
|  |  |  |  | all else is kept |
|  | Sepsis | | PRSEPIS | "NULL" is removed |
|  |  |  |  | "Sepsis" is removed |
|  |  |  |  | "Septic Shock" is removed |
|  |  |  |  | "SIRS" is removed |
|  |  |  |  | "None" is kept |
|  | Pneumonia | | CPNEUMON | "NULL" is removed |
|  |  |  |  | "Yes" is removed |
|  |  |  |  | "No" is kept |
|  | Wound infection | | WNDINF | "Yes" is removed |
|  |  |  |  | "No" is kept |
|  | Disseminated cancer | | DISCANCR | "Yes" is removed |
|  |  |  |  | "No" is kept |
|  | Renal failure | | RENAFAIL | "Yes" is removed |
|  |  |  |  | "No" is kept |
|  | Chronic steroid use | | STEROID | "Yes" is removed |
|  |  |  |  | "No" is kept |
| **Preoperative** | Leukocytosis | | PRWBC | >=11 = "Yes" |
|  |  |  |  | <11 = "No" |
|  |  |  |  | "-99" is already removed |
|  | Age | | Age | "90+" = 90 |
|  |  |  |  | No modification to all else |
|  | Creatinine | | PRCREAT | "-99" is removed |
|  |  |  |  | No modification to all else |
|  | Sex | | SEX | "NULL" is removed |
|  |  |  |  | "male" = "male" |
|  |  |  |  | "female" = "female" |
|  | ASA grade | | ASACLAS | "None assigned" is removed |
|  |  |  |  | "NULL" is removed |
|  |  |  |  | "1-No Disturb" = "ASA less than 3" |
|  |  |  |  | "2-Mild Disturb" = "ASA less than 3" |
|  |  |  |  | "3-Severe Disturb" = "ASA 3 or greater" |
|  |  |  |  | "4-Life Threat" = "ASA 3 or greater" |
|  |  |  |  | "5-Moribund" = "ASA 3 or greater" |
|  | Obesity | | WEIGHT, HEIGHT | "-99" in either variable is removed |
|  |  |  |  | 703*WEIGHT/HEIGHT^2>30 = "BMI>30" |
|  |  |  |  | 703*WEIGHT/HEIGHT^2<=30 = "No Complication" |
|  | Diabetes | | DIABETES | "INSULIN" = "Yes" |
|  |  |  |  | "NON-INSULIN"= "Yes" |
|  |  |  |  | "ORAL" = "Yes" |
|  |  |  |  | "No" = "No" |
|  | Hypertension | | HYPERMED | "Yes" = "Yes" |
|  |  |  |  | "No" = "No" |
|  | Tobacco smoker | | SMOKE | "Yes" = "Yes" |
|  |  |  |  | "No" = "No" |
|  | Alcohol consumption | | ETOH | "Yes" = "Yes" |
|  |  |  |  | "No" = "No" |
|  | Dyspnea | | DYSPNEA | "AT REST" = "Yes" |
|  |  |  |  | "MODERATE EXERTION" = "Yes" |
|  |  |  |  | "No" = "No" |
|  | Angina | | HXANGINA | "Yes" = "Yes" |
|  |  |  |  | "No" = "No" |
|  | Emergency procedure | | EMERGNCY | "Yes" = "Yes" |
|  |  |  |  | "No" = "No" |
|  | History of COPD | | HXCOPD | "Yes" = "Yes" |
|  |  |  |  | "No" = "No" |
|  | History of chronic heart failure | | HXCHF | "Yes" = "Yes" |
|  |  |  |  | "No" = "No" |
|  | History of myocardial infarction | | HXMI | "NULL" is removed |
|  |  |  |  | "Yes" = "Yes" |
|  |  |  |  | "No" = "No" |
|  | History of PVD | | HXPVD | PRVPCS\|PRVPCI="Yes" = "Yes" |
|  |  |  |  | PRVPCS&PRVPCI="No" = "No" |
|  | Previous cardiac procedure | | PRVPCS, PRVPCI | PRVPCS\|PRVPCI="Yes" = "Yes" |
|  |  |  |  | PRVPCS&PRVPCI="No" = "No" |
| **Intraoperative** | Operation time | | OPTIME | "-99" is removed |
|  |  |  |  | No modification to all else |
|  | Blood Transfusions | | OTHBLEED | "Transfusions/Intraop/Postop" = "Transfusions/Intraop/Postop" |
|  |  |  |  | "Bleeding/Transfusions" = "Transfusions/Intraop/Postop" |
|  |  |  |  | "No Complication" = "No Complication" |
|  | Procedure | | CPT | 33361-33478 = "Valvular" |
|  |  |  |  | 33510-33548 = "CABG" |
|  |  |  |  | Any combination of valvular and CABG = "CABG" |
|  |  |  |  | All else = "Other" |
| **Postoperative (within 30 days)** | 30-day mortality | | DOpertoD, DISCHDEST | 0<=DOpertoD<=30\|DISCHDEST="Expired" = "Yes" |
|  |  |  |  | DOpertoD=-99&DISCHDEST!="Expired" = "No" |
|  | Wound Complication (composite) | Superficial surgical site infection | SUPINFEC | "Superficial Incisional SSI" = "Superficial Incisional SSI" |
|  |  |  |  | "No Complication" = "No Complication" |
|  |  | Deep wound infection | WNDINFD | "Deep Incisional SSI" = "Deep Incisional SSI" |
|  |  |  |  | "No Complication" = "No Complication" |
|  |  | Organ space infection | ORGSPCSSI | "Organ/Space SSI" = "Organ/Space SSI" |
|  |  |  |  | "No Complication" = "No Complication" |
|  |  | Wound disruption | DEHIS | "Wound Disruption" = "Would Disruption" |
|  |  |  |  | "No Complication" = "No Complication" |
|  | Medical Complication (composite) | Unplanned reintubation | REINTUB | "Unplanned Intubation" = "Unplanned Intubation" |
|  |  |  |  | "No Complication" = "No Complication" |
|  |  | Pulmonary embolism | PULEMBOL | "Pulmonary Embolism" = "Pulmonary Embolism" |
|  |  |  |  | "No Complication" = "No Complication" |
|  |  | Need for ventilator >48 hours | FAILWEAN | "On Ventilator greater than 48 Hours" = "On Ventilator greater than 48 Hours" |
|  |  |  |  | "No Complication" = "No Complication" |
|  |  | Renal insufficiency | RENAINSF | "Progressive Renal Insufficiency" = "Progressive Renal Insufficiency" |
|  |  |  |  | "No Complication" = "No Complication" |
|  |  | Renal failure | OPRENAFL | "Acute Renal Failure" = "Acute Renal Failure" |
|  |  |  |  | "No Complication" = "No Complication" |
|  |  | Urinary tract infection | URNINFEC | "Urinary Tract Infection" = "Urinary Tract Infection" |
|  |  |  |  | "No Complication" = "No Complication" |
|  |  | Stroke | CNSCVA | "Stroke/CVA" = "Stroke/CVA" |
|  |  |  |  | "No Complication" = "No Complication" |
|  |  | Coma | CNSCOMA | "Coma greater than 24 hours" = "Coma greater than 24 hours" |
|  |  |  |  | "Coma greater than 24 Hours" = "Coma greater than 24 hours" |
|  |  |  |  | "No Complication" = "No Complication" |
|  |  | Cardiac arrest | CDARREST | "Cardiac Arrest Requiring CPR" = "Cardiac Arrest Requiring CPR" |
|  |  |  |  | "No Complication" = "No Complication" |
|  |  | Myocardial infarction | CDMI | "Myocardial Infarction" = "Myocardial Infarction" |
|  |  |  |  | "No Complication" = "No Complication" |
|  |  | Pneumonia | OUPNEUMO | "Pneumonia" = "Pneumonia" |
|  |  |  |  | "No Complication" = "No Complication" |
|  |  | Deep vein thrombosis | OTHDVT | "DVT Requiring Therapy" = "DVT Requiring Therapy" |
|  |  |  |  | "DVT/Thrombophlebitis" = "DVT Requiring Therapy" |
|  |  |  |  | "No Complication" = "No Complication" |
|  |  | Sepsis | OTHSYSEP | "Sepsis" = "Sepsis" |
|  |  |  |  | "No Complication" = "No Complication" |
|  |  | Septic shock | OTHSESHOCK | "Septic Shock" = "Septic Shock" |
|  |  |  |  | "No Complication" = "No Complication" |
